# Productivity Index in Clinical Andrology: Research Directions on High-Impact Topics and in Particular on Male Infertility

**DOI:** 10.3390/jcm12093152

**Published:** 2023-04-27

**Authors:** Andrea Crafa, Aldo Eugenio Calogero, Rossella Cannarella, Rosita Angela Condorelli, Antonio Aversa, Sandro La Vignera

**Affiliations:** 1 Department of Clinical and Experimental Medicine, University of Catania, 95123 Catania, Italy; 2 Glickman Urological & Kidney Institute, Cleveland Clinic, Cleveland, OH 44195, USA; 3 Department of Experimental and Clinical Medicine, University Magna Graecia of Catanzaro, 88100 Catanzaro, Italy

**Keywords:** productivity index, andrology, male infertility, andrological field, top scientist

## Abstract

Purpose: Andrological diseases have an important social and economic impact as they cause a serious impairment of the quality of life of the affected patient. Epidemiologically, the impact of these disorders is progressively increasing, as demonstrated by the ever-growing prevalence of male infertility. This evidence justifies the rapid development of research in andrology that the scientific community has undertaken in recent decades. This study aims to evaluate the productivity index of the main andrological topics studied and reported in the literature. Methods: The total number of published articles was extracted from the Scopus database by entering the following keywords and mesh terms: “Male Infertility”, “Erectile Dysfunction”, “Premature Ejaculation”, “Male Hypogonadism”, “Testicular Tumors”, “Prostate Cancer”, “Prostatic hyperplasia”, “Prostate hyperplasia”, “Prostatitis”, “Prostate inflammation”, and “Male Accessory Gland Infections”. Furthermore, a list of the top 50 researchers sorted by productivity was created for each topic. For male infertility, a further search was performed by combining the search term “male infertility” with the above-mentioned terms. Thus, a list of the top 30 authors in order of productivity was also extracted. The graphs were created using Excel. Results and Conclusions: As could be expected, we observed that prostate cancer and male infertility were the two most investigated topics, followed by benign prostatic hyperplasia and erectile dysfunction, whose prevalence is set to increase given the progressive aging of the population. Less investigated is the inflammation of the accessory sexual glands. In conclusion, this study provides a ranking of the main andrological topics investigated in the literature, also presenting the top list of the most productive authors for each one.

## 1. Introduction

Endocrine disorders represent an important cause of morbidity and mortality that significantly impact quality of life. In particular, among endocrine diseases, disorders of gonadal function have an epidemiological impact second only to that of metabolic disorders [1]. It is also interesting to consider that the latter, such as diabetes mellitus (DM) and obesity, are often associated with andrological dysfunctions. For example, the association between DM and erectile dysfunction (ED) is well known, with approximately half of all diabetic patients complaining of ED [2]. Likewise, obesity has been related to male hypogonadism and subfertility in both men and women [3]. Furthermore, infertility is a widespread disease, which weighs heavily on modern society. Over the years, in fact, there has been a slow continuous decline in the quality of sperm parameters [4]. It is therefore not surprising that nearly 186 million people worldwide are infertile [5], and the cause of infertility can be attributed to the man in about half of the cases. However, in approximately 30–50% of cases, the cause of male infertility is still unknown (idiopathic infertility) [6]. This evidence justifies the rapid development of research in the field of andrology that the scientific community has undertaken in recent decades. Furthermore, andrology is experiencing a “renaissance”, with a growing interest in the genetic and epigenetic aspects of infertility and other andrological diseases. Furthermore, the use of artificial intelligence methods to study neoplastic diseases, such as prostate and testicular tumors, is another example of how andrological research has evolved in recent years [7]. Born as a branch of endocrinology or urology, today, andrology represents a fully developed discipline, which is destined to grow further. This has required the efforts of many scientists from all over the world. Therefore, this study aimed to evaluate the productivity index, i.e., the quantity of articles produced on a certain theme, of the main andrological topics studied and reported in the literature. Given the impact of male infertility, more research needs to be carried out on various subtopics related to this field.

## 2. Materials and Methods

Since the field of andrology covers diverse and wide-ranging topics, we have decided to focus our attention on diseases with the highest prevalence and the greatest impact on the quality of life of affected patients.

Therefore, a thorough search was performed on the Scopus database by entering the following keywords and mesh terms: “Male Infertility”, “Erectile Dysfunction”, “Premature Ejaculation”, “Male Hypogonadism”, “Testicular Tumors”, “Prostate Cancer”, “Prostatic hyperplasia”, “Prostate hyperplasia”, “Prostatitis”, “Prostate inflammation”, and “Male Accessory Gland Infections”.

For these topics, the total number of published articles was evaluated, and then, using the Scopus function that allows the subdivision of articles by author, the list of the top 50 researchers sorted by productivity was extracted. As far as male infertility is concerned, a further search was carried out by inputting combinations of the following keywords: “Male infertility AND varicocele”, “Male infertility AND male hypogonadism”, “Male infertility AND diabetes”, “Male infertility AND prostatitis”, “Male infertility AND prostate inflammation”, “Male infertility AND male accessory gland infection”, “Male infertility AND testicular cancer”, “Male infertility AND sperm DNA fragmentation”, “Male infertility AND FSH treatment”, and “Male infertility AND obesity”. As for the first search, the total number of articles was evaluated, but in this case, a list of the top 30 authors in order of productivity was extracted. The graphs were created using Excel.

## 3. Results

**Figure 1:**
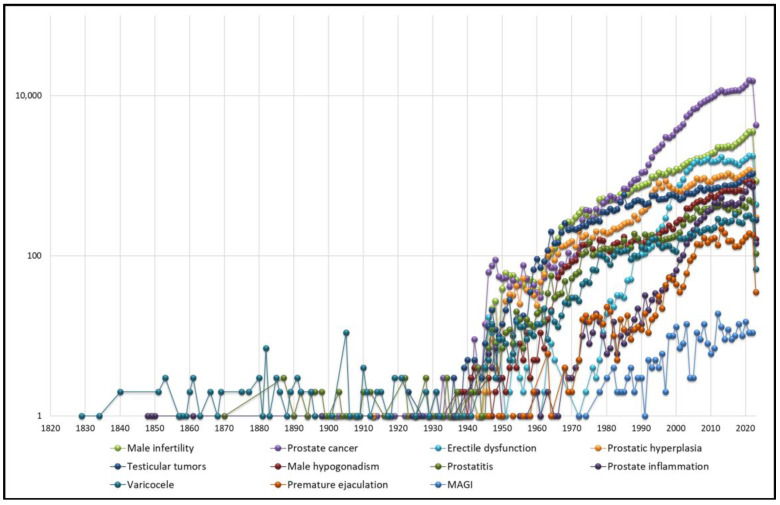
Number of publications over the years from the main andrological areas of study.

The list of the main andrological topics in order of productivity is shown in Table 1. From Figure 1, it can be seen that publications in various andrological topics have grown significantly over the last 60 years. In particular, prostate cancer, although it has always been the most studied topic, has seen a remarkable increase in the number of publications compared to other areas in the last 20 years.

Immediately after prostate cancer, the topic that would seem to be most investigated is male infertility, which, unlike other topics (e.g., varicocele and prostatitis) has its origins in more recent research, but for which interest from the scientific community has grown exponentially.

The form of male sexual dysfunction that has seen the highest number of publications is erectile dysfunction, which is also the third most investigated topic. However, it is possible to observe that in the last 10 years, there has been a rapid growth in the number of publications compared to the past 20 years. Conversely, premature ejaculation is one of the least investigated andrological topics.

A similar trend of rapid growth over the past 20 years can also be observed for male hypogonadism, which ranks sixth on this list. The inflammatory aspect, on the other hand, although it is one the topics that was studied in the past, when andrology was not yet considered a branch with its own identity, has been less investigated, with only 13,042 articles found when the term “prostatitis” was input and even fewer when the terms “prostatic inflammation” and “male accessory gland infections (MAGI)” (Table 1) were used. It is also interesting to note that varicocele is also a topic that has been investigated for a long time but has acquired new value in the last 30 years.

The analysis of the researchers in order of productivity in terms of individual topics is shown in Figure 2, Figure 3, Figure 4, Figure 5, Figure 6, Figure 7, Figure 8, Figure 9, Figure 10, Figure 11 and Figure 12.

**Figure 13:**
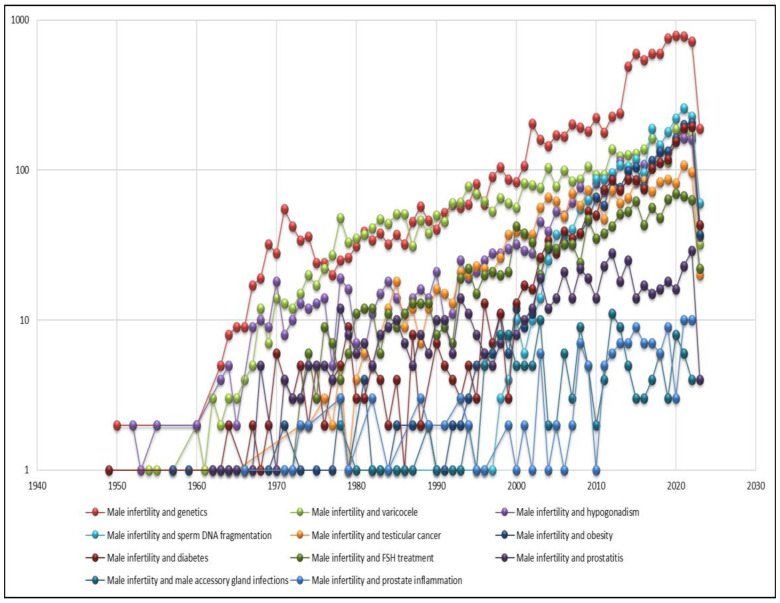
Variation in the number of publications over the years on male infertility and other popular subtopics.

Regarding the sub-research on male infertility, we observed that the largest number of articles was concerned with the association between male infertility and genetics. In particular, it can be seen from Figure 13 that genetic research in fertility is of fairly recent origin, but in the last two decades especially, and even more in the last 10 years, it has seen a remarkable growth in the number of publications. In second place is varicocele, which despite being among the least investigated andrological topics has been much studied in association with infertility. Another well-studied association is between male infertility and sperm DNA fragmentation (SDF). In particular, the first studies on the SDF rate in infertility were published in the 1980s, with rapid and exponential growth in the number of publications starting from the 2000s and in the last decade particularly.

In fourth place were publications on male infertility and testicular tumors, followed by publications that correlated the main metabolic diseases, DM and obesity, to infertility. In particular, for these last two subtopics, interest has increased especially in recent years and research trends seem to demonstrate that the interest in the association between dysmetabolism and infertility will progressively increase. Again, the association between infertility and inflammation has been studied the least (Table 2).

The analysis of the researchers in order of productivity on the individual topics of this sub-research is shown in Figure 14, Figure 15, Figure 16, Figure 17, Figure 18, Figure 19, Figure 20, Figure 21, Figure 22, Figure 23 and Figure 24.

## 4. Discussion

Despite the significant impact of andrological diseases and although male infertility is a global health problem, considerable progress is still needed regarding the management of these diseases, especially in the area of male reproductive health [8]. This article aimed to analyze the productivity index on the main andrological topics, provide an overview of the research in this field to date, and offer insights into what still needs to be done. In particular, our research has shown that the most investigated topic is certainly prostate cancer. This is not surprising given the high prevalence of this cancer, which is the third most common cancer after breast and lung cancer and the second most common cancer in men. It is also the fifth leading cause of death globally [9]. Thanks to advances in diagnostics and treatment, the management of patients with prostate cancer have greatly improved over the years [8]. Furthermore, the growing understanding of the biological and genetic mechanisms underlying the genesis of this tumor will allow for the ever-better diagnostic definition and therapeutic management of the disease [10]. Furthermore, the rapid growth of research in the last twenty years is probably attributable to the improvement of diagnostic imaging techniques and, in particular, of multiparametric magnetic resonance (mpRMI), which has allowed an adequate study of the disease, improving its staging, allowing targeted biopsies and the adequate active surveillance of the less aggressive forms [11]. Furthermore, in the last few years, a new thrust of research can probably be attributed to the extensive use of artificial intelligence applied to the study of mpRMI images [7].

Our research strategy identified male infertility as the second most investigated topic in andrology. This is likely due to the significant global impact of infertility, as mentioned above. In particular, the rapid growth of research in the field of male infertility would appear to correlate directly with the rapid improvement in the genetic study of this condition. In fact, 1/6 of the publications on male infertility seem to be related to genetic aspects, which therefore appear to be the most investigated subtopics. This is probably due to the introduction of next-generation sequencing techniques in the last twenty years, which have made it possible to identify a large percentage of genes involved in the pathogenesis of non-obstructive azoospermia and infertility hitherto identified as idiopathic [12,13]. Probably also contributing is the growing body of evidence correlating male infertility with men’s general health statuses that focuses on the relationship between male infertility and oncologic, cardiovascular, autoimmune, and metabolic diseases [14]. Regarding the latter, our research has shown that the relationships between male infertility and metabolic diseases, such as DM and obesity, are among the most investigated. This is not surprising given the high epidemiological impact of these two conditions. DM is a disease with a significant epidemiological impact. Indeed, more than 537 million people between the ages of 20 and 79 (10.5 percent of the world’s population) suffer from it, according to the International Diabetes Federation [15]. DM would appear to be able to influence fertility through several mechanisms that are different in patients with type 1 DM as opposed to type 2 DM. Indeed, in the first type, the volume of ejaculated seminal fluid appears to be altered, probably due to the contractile dysfunction of the epididymis and seminal vesicles due to diabetic neuropathy. Sperm motility also appears to be compromised due to mitochondrial damage [16]. Confirming this, meta-analytic data showed low sperm motility in type 1 DM patients and a trend toward low seminal volume. Furthermore, sperm morphology would also appear to be altered in these patients [17]. In type 2 DM, on the other hand, the inflammatory microenvironment induced by the disease would be at the basis of an increase in oxidative stress with the consequent alteration of seminal parameters and DNA fragmentation [16]. However, further studies are needed to better clarify the mechanisms involved and the impact of DM on fertility. This explains the great interest in scientific research in this regard. In terms of obesity, more than 1.9 billion adults are overweight, and a third of these are obese (about 13% of the world’s population). However, even more staggering is that over 340 million children and adolescents between the ages of 5 and 19 were overweight or obese in 2016 [18]. This finding may be one of the factors involved in the decline in sperm quality in adults, as rising rates of obesity have been associated with declines in sperm function over the years [19].

The association between tumor and infertility is another close relationship that we found. In particular, it is known that the treatments that are carried out to cure neoplastic diseases damage fertility [10]. However, for testicular tumors, there is evidence in favor of an association between altered sperm parameters and an increased prevalence of testicular tumors. This association is of great importance, considering that testicular tumors are the most common solid tumors in men aged 14 to 44 years [20]. Based on the association between the two diseases, testicular dysgenesis syndrome is apparently present, which in turn can be attributed to lifestyle and environmental changes that cause exposure to endocrine disruptors during fetal life, with the consequent impairment of correct fetal development. This is another condition that helps explain the decline in fertility observed over the past 50 years [21]. Considering the epidemiological impact on the young fertile population, it is not surprising that our research has shown that the testicular tumor is the 5th most investigated andrological topic in general and the 4th most researched in association with male infertility.

In the context of male infertility, we found that the most studied association is with varicocele. In fact, although in general the varicocele topic is among the least investigated in the andrological field, most of the publications that include it studied its relationship in association with male infertility. Not by chance, varicocele is considered the most common correctable cause of male infertility, and a recent meta-analysis confirmed that varicocele repair is able to improve conventional sperm parameters compared to untreated controls [22]. The extensive scientific research on this topic has also led to the understanding that not all varicoceles need to be treated but that only patients with clinically significant varicoceles associated with a documented history of infertility and altered sperm parameters can benefit from treatment. Conversely, the current available evidence does not seem to support the benefit of treating a subclinical varicocele or a bilateral varicocele that is not clinically significant [23]. Considering that varicocele is present in about 35% of patients with primary infertility and the controversy still present regarding its therapeutic management in the context of infertility [24], it is not surprising that the association between male infertility and varicocele is still being extensively investigated.

Our search strategy subsequently highlighted that hypogonadism, another condition closely associated with infertility, has been extensively studied. Indeed, hypogonadism has a considerable impact on the quality of life of the individual and on fertility, as suggested by the fact that in 75% of infertility cases, a primary testicular dysfunction is present [25]. However, in the last two decades, the classification of male hypogonadism has changed, with the genetic forms playing only a small pathogenetic role. In fact, the birth of the definition of late-onset hypogonadism [26] and metabolic hypogonadism has changed the vision of this condition [27]. Indeed, preclinical and clinical evidence seems to confirm that late-onset hypogonadism and metabolic dysfunctions are closely related to each other and that there is a reciprocal influence between the two conditions [28]. Furthermore, dysmetabolic diseases, both indirectly through the reduction of FSH and testosterone levels and by acting directly on spermatozoa, could compromise reproductive potential, although further studies are needed to better investigate this aspect [29].

Our results also noted that among the most studied associations was that between male infertility and SDF. Consequently, the extensive literature that has been published over the years led the World Health Organization in 2021 to introduce the SDF test as a test to be requested under certain circumstances for a better assessment of the fertility status, giving this test a diagnostic value and not only value in scientific research [30]. However, to date, there is still no consensus on which patients would benefit most from this diagnostic investigation and even scientific societies provide little guidance in this regard [31]. This is exactly why efforts are being made to identify which parameters can best predict which patients should be investigated for the presence of SDF. In this context, a recent study has shown that the combination of unexplained infertility, multiple abortion history, testicular volume < 15 (according to the Prader’s orchidometer), age ≥ 38 years, and a total motile sperm count of < 20 × 10^6^ would seem to be a model capable of predicting with high accuracy the presence of SDF. All of this evidence explains why SDF is a hot topic in andrology today [32].

Much less investigated are the inflammatory aspects and their impact on fertility. In our opinion, this result is quite surprising given the high prevalence of inflammatory diseases on the male population. Indeed, 5–40% of men suffer from MAGI [33]. Furthermore, the evidence suggests that more extensive forms of MAGI with simultaneous involvement of the prostate, epididymis, and seminal vesicles would be more detrimental to semen quality than prostatitis alone [33]. Similarly, the correct diagnostic definition of these inflammatory conditions and their early treatment would prevent the development of sclero-atrophic forms of prostate-vesicular-epididymitis that are associated with irreversible damage to seminal quality [33]. Finally, evidence also suggests the association of inflammatory disease with the development of benign prostatic hyperplasia (BPH), sexual dysfunction (ED), and premature ejaculation and even more severe conditions, such as more aggressive forms of prostate cancer [34]. Thus, we suggest that the inflammatory disease of the male accessory sex glands should receive more attention from the point of view of the scientific research.

Finally, our results showed wide attention to scientific research on two other highly prevalent andrological conditions: BPH and ED. Regarding the former, attention from the scientific community is not surprising, since BPH has a very large impact on the quality of life of the patients who suffer from it. In fact, in 2017, it was estimated that the years lived with disease attributed to BPH/lower urinary tract syndrome were 2,247,334, compared to 843,226 for prostate cancer. This impact is expected to increase considering the progressive aging of the general population [35]. Similarly, ED has an important social and economic impact. Patients with ED, indeed, have a worse quality of life, higher rates of depression and anxiety, and reduced productivity at work. Furthermore, the quality of life of partners of ED patients is also significantly affected [36]. It also correlates significantly with the presence of other sexual dysfunctions, such as premature ejaculation [37] and low sexual desire [38]. Moreover, the correlation between the presence of erectile dysfunction and cardiovascular disease has been known for some time, increasing the interest in this topic [39]. As a consequence, the high prevalence of ED led to a growing effort to identify new treatments that result in benefits for affected patients. This seems to explain why research on the topic has grown exponentially in the last 20 years. To date, the main interventions consist of oral therapy, intracavernous injections, vacuum devices, and penile prostheses. However, the roles of stem cells or penile low-intensity shock wave lithotripsy are also much studied today, and the use of gene therapies is also envisioned in the future [40].

Our study has some limitations. First, the search was performed on a single database, although Scopus was the database that produced the most results after keyword entry compared to others, such as PubMed or Web of Science [41]. Another limitation is the fact that the results were not screened. Therefore, there is the possibility that some articles were not entirely relevant to the searched topic. However, using Scopus’s TITLE-ABS-KEY function makes it likely that only articles with content relevant to specific keywords were selected. Finally, not all andrological topics and subtopics were evaluated. However, the aim of our study was to analyze those with the highest epidemiological impact.

## 5. Conclusions

Andrological diseases have a significant social and economic impact and compromise the quality of life of affected patients. Undoubtedly, the extensive research in this area has led, over the years, to a better understanding of the pathophysiological mechanism of these diseases and the clinical management of patients suffering from them. However, much remains to be done. Indeed, the progressive increase in age will lead to an ever-higher prevalence of some of these diseases (e.g., prostate cancer, BPH, ED, etc.). Similarly, for male infertility, much remains to be understood about the pathophysiological mechanisms underlying this condition and its correct management. Surprisingly, the inflammatory diseases of the male accessory glands appear to be less explored, despite their impact on the quality of life of affected patients and other associated andrological conditions. Our study provides a global overview of andrological research to date, revealing which topics are most investigated and which authors have contributed most to the research on each topic.

## Figures and Tables

**Figure 2:**
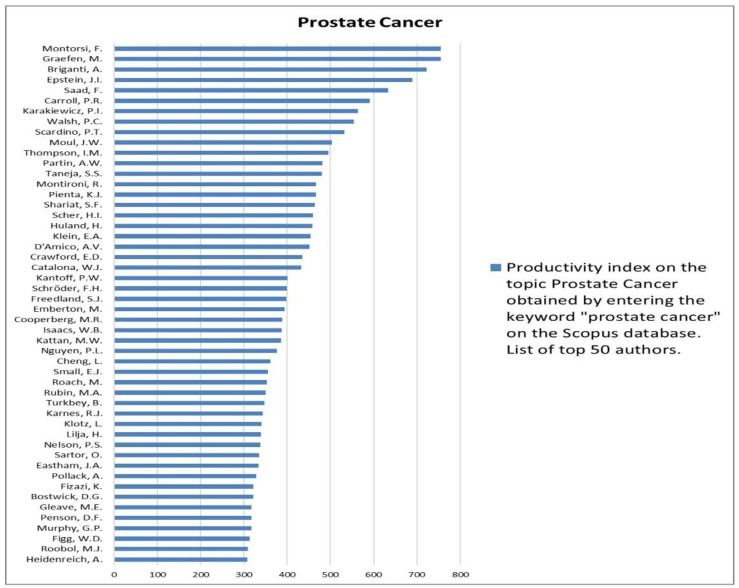
List of top 50 authors in terms of productivity in the area of prostate cancer.

**Figure 3:**
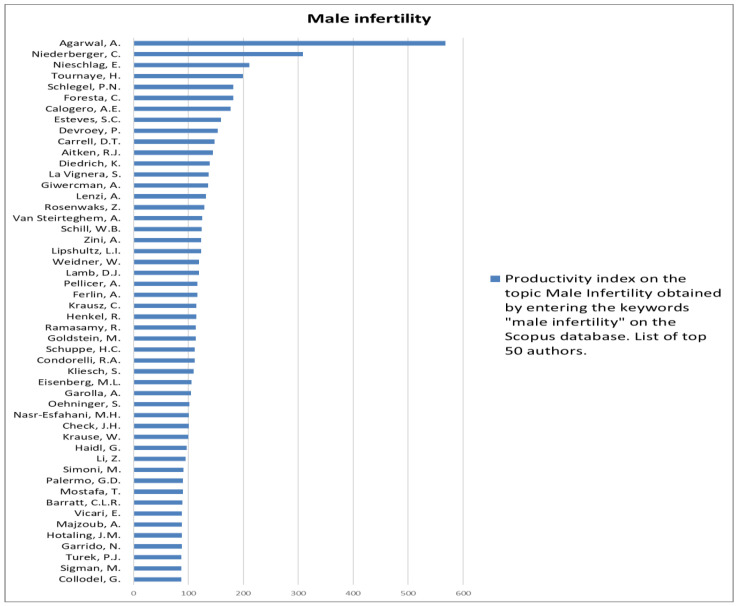
List of top 50 authors in terms of productivity in the area of male infertility.

**Figure 4:**
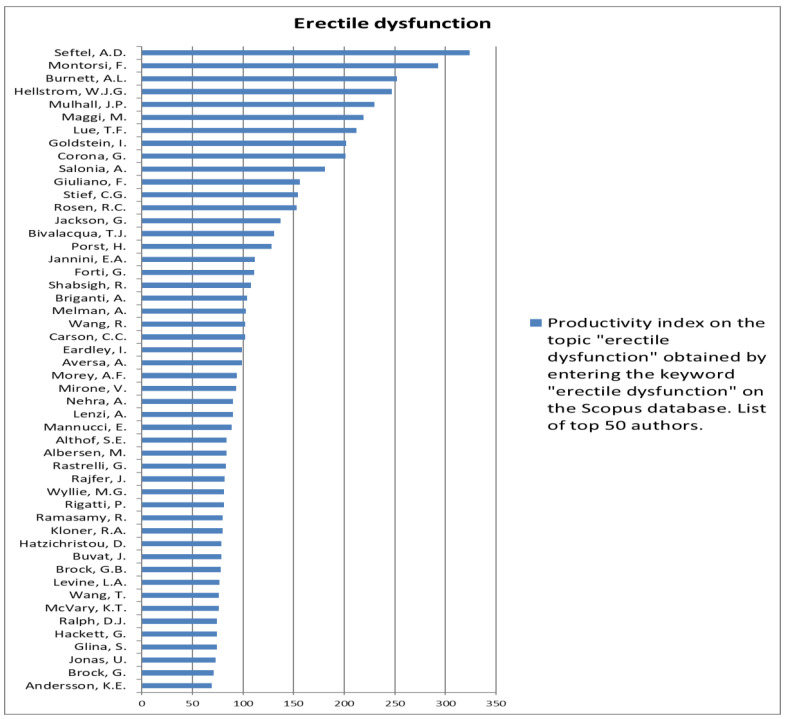
List of top 50 authors in terms of productivity in the area of erectile dysfunction.

**Figure 5:**
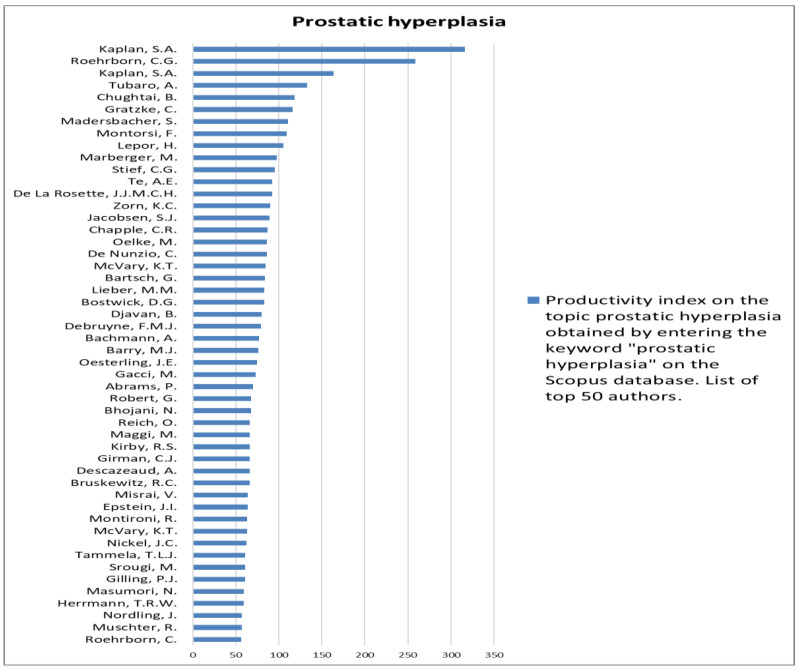
List of top 50 authors in terms of productivity in the area of prostatic hyperplasia.

**Figure 6:**
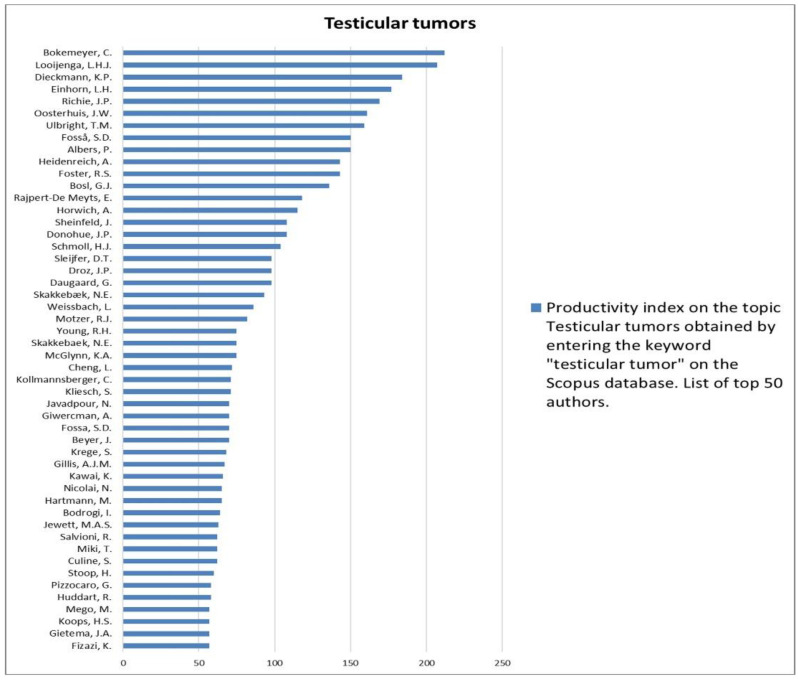
List of top 50 authors in terms of productivity in the area of testicular tumors.

**Figure 7:**
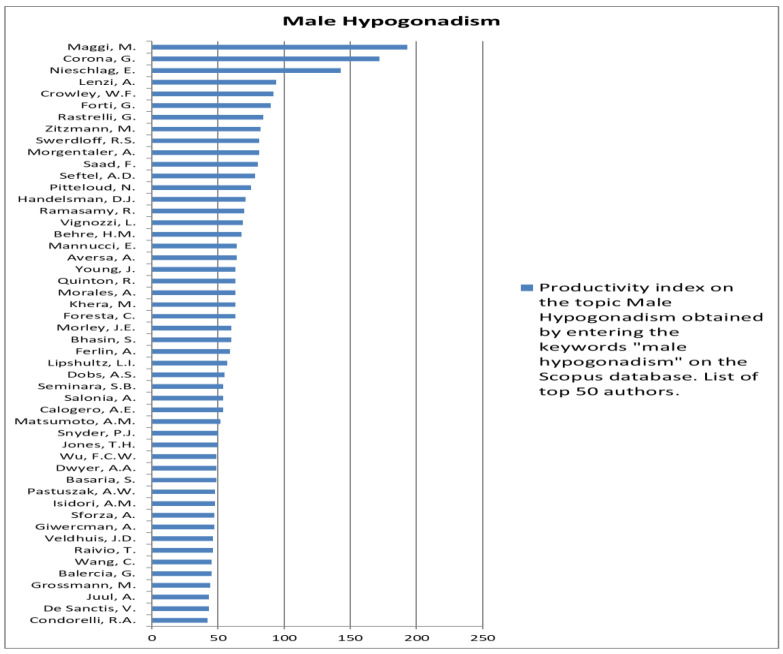
List of top 50 authors in terms of productivity in the area of male hypogonadism.

**Figure 8:**
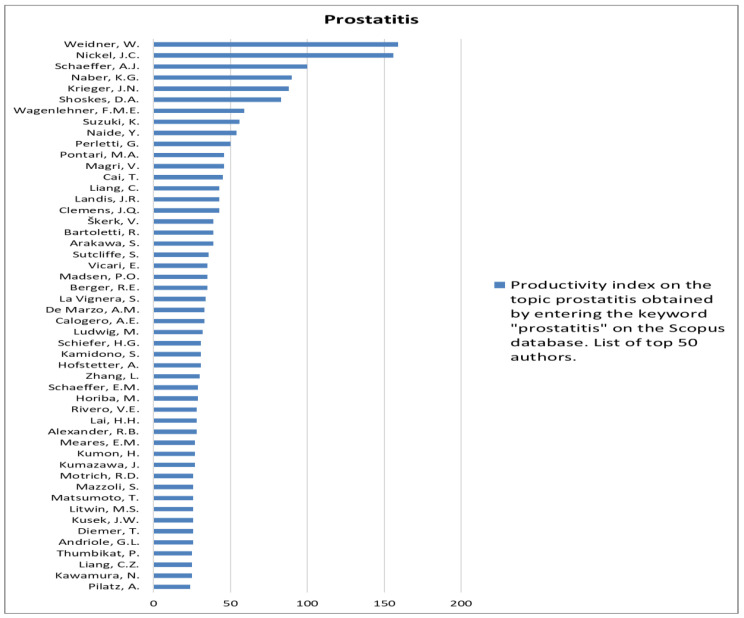
List of top 50 authors in terms of productivity in the area of prostatitis.

**Figure 9:**
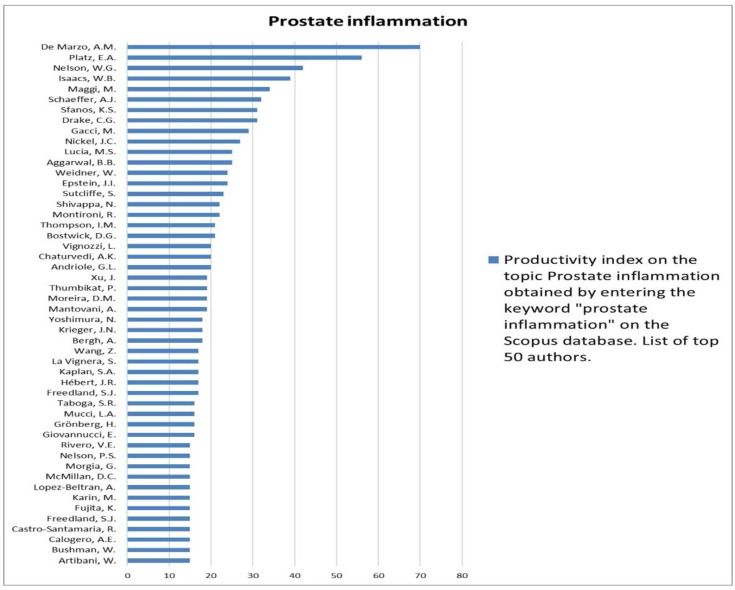
List of top 50 authors in terms of productivity in the area of prostate inflammation.

**Figure 10:**
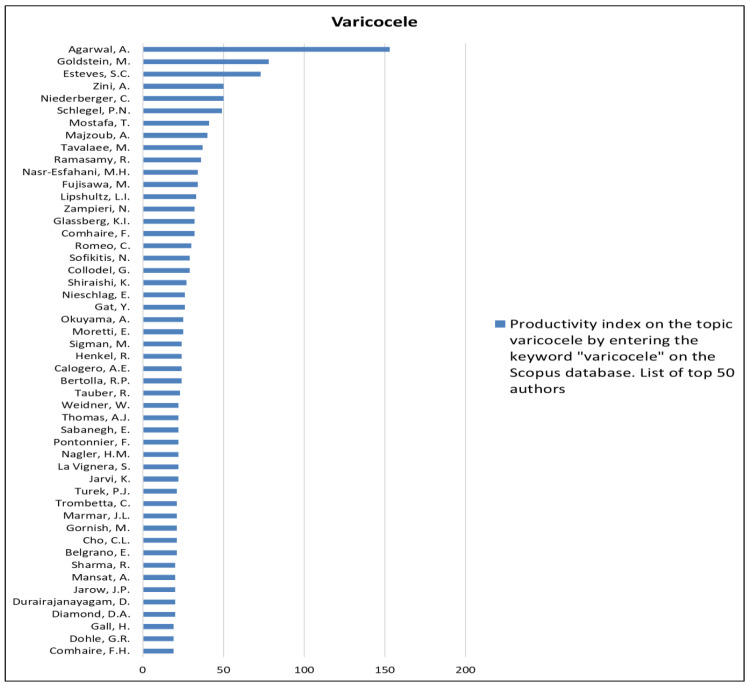
List of top 50 authors in terms of productivity in the area of varicocele.

**Figure 11:**
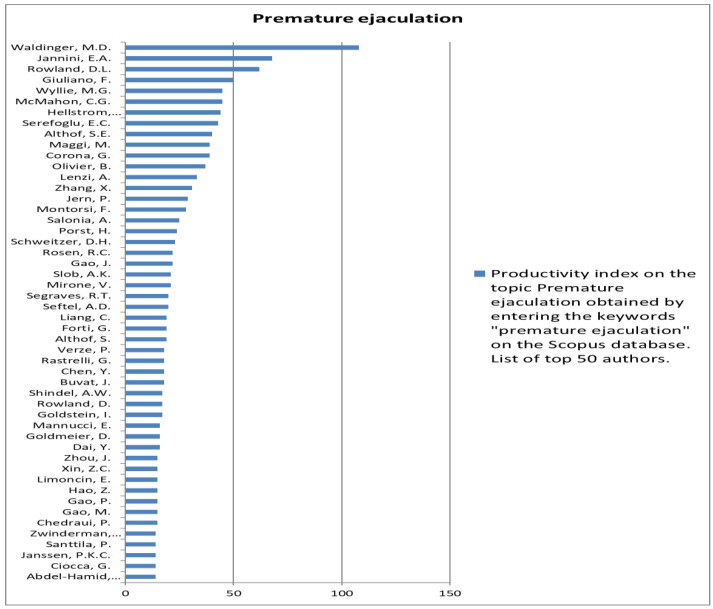
List of top 50 authors in terms of productivity in the area of premature ejaculation.

**Figure 12:**
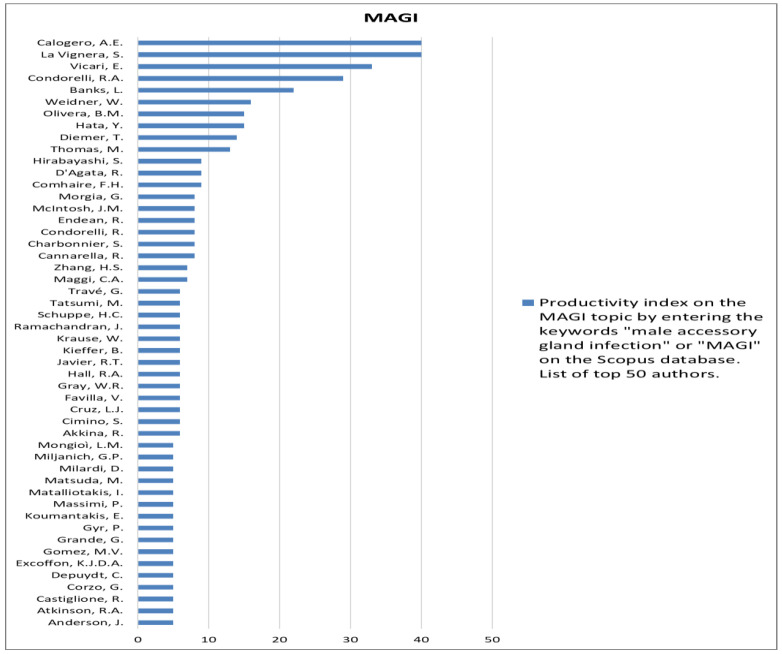
List of top 50 authors in terms of productivity in the area of male accessory gland infection.

**Figure 14:**
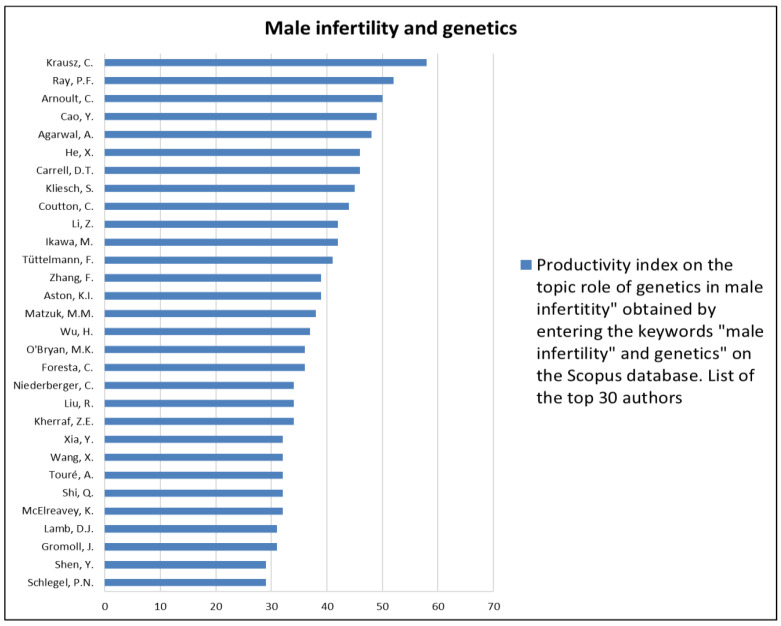
List of top 30 authors in terms of productivity regarding the association between male infertility and genetics.

**Figure 15:**
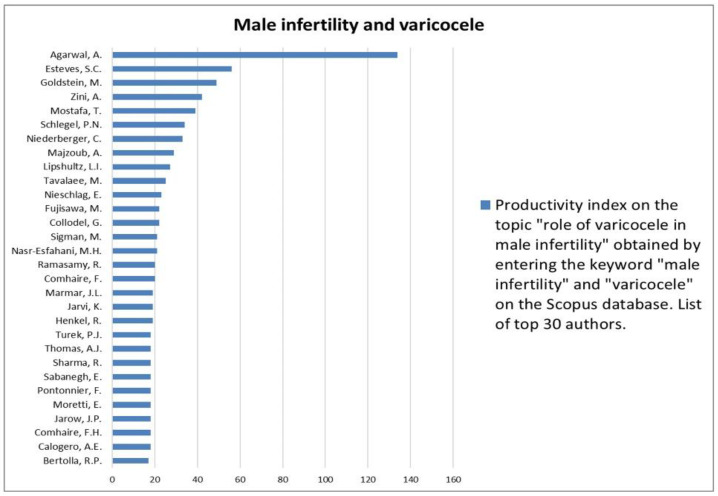
List of top 30 authors in terms of productivity regarding the association between male infertility and varicocele.

**Figure 16:**
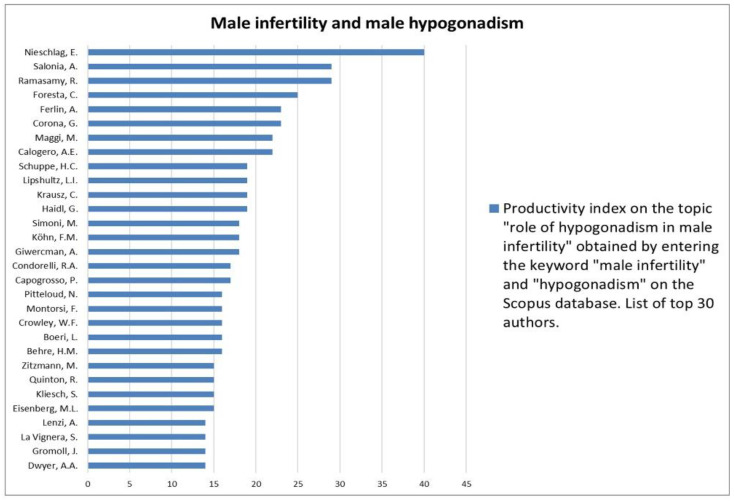
List of top 30 authors in terms of productivity regarding the association between male infertility and male hypogonadism.

**Figure 17:**
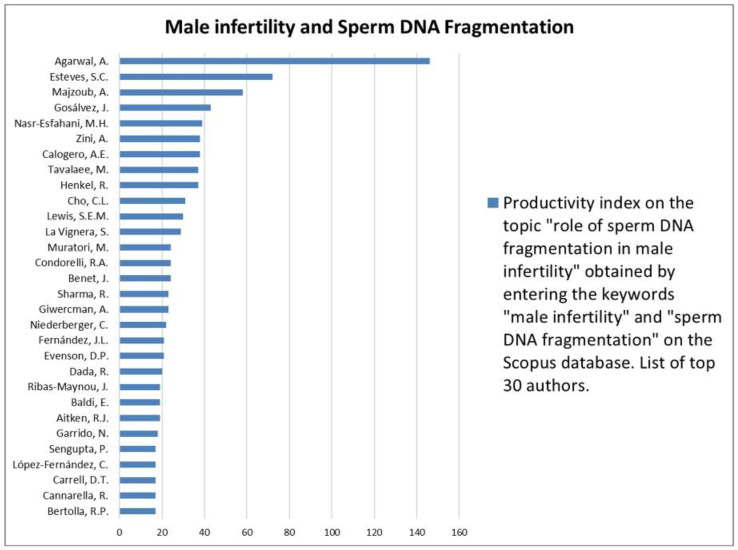
List of top 30 authors in terms of productivity regarding the association between male infertility and sperm DNA fragmentation.

**Figure 18:**
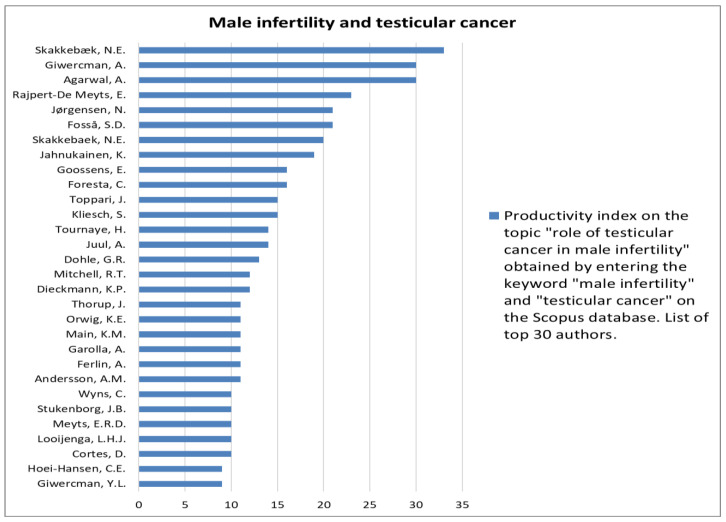
List of top 30 authors in terms of productivity regarding the association between male infertility and testicular cancer.

**Figure 19:**
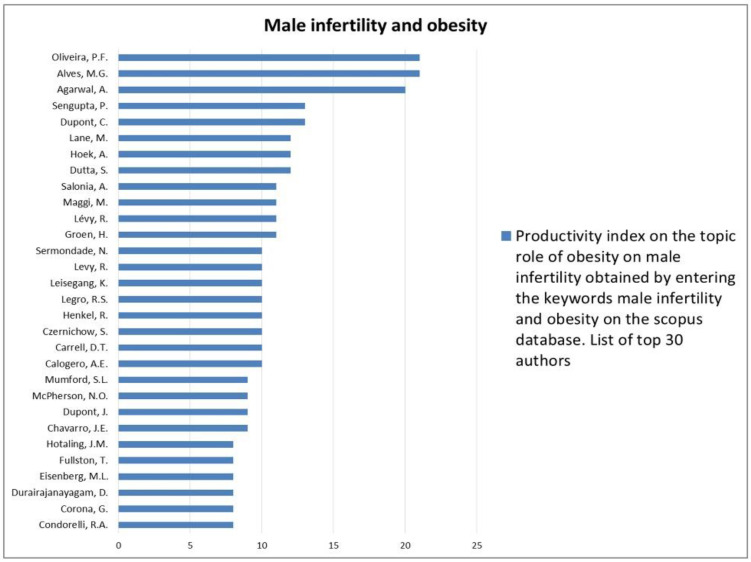
List of top 30 authors in terms of productivity regarding the association between male infertility and obesity.

**Figure 20:**
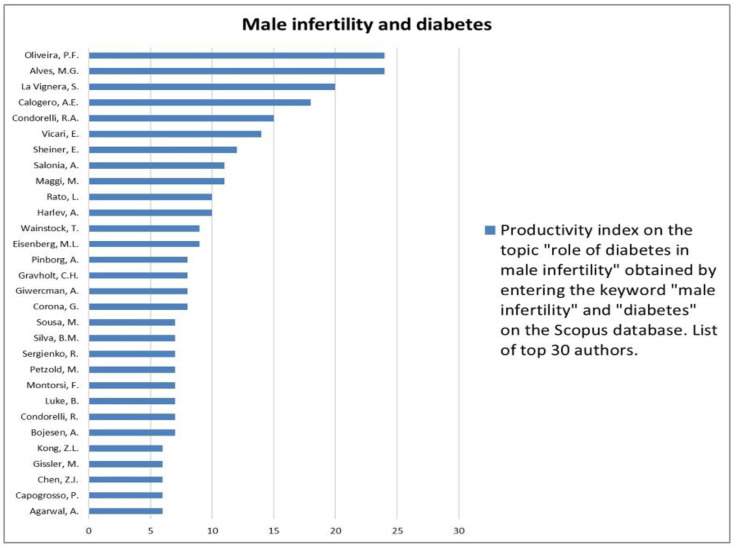
List of top 30 authors in terms of productivity regarding the association between male infertility and diabetes.

**Figure 21:**
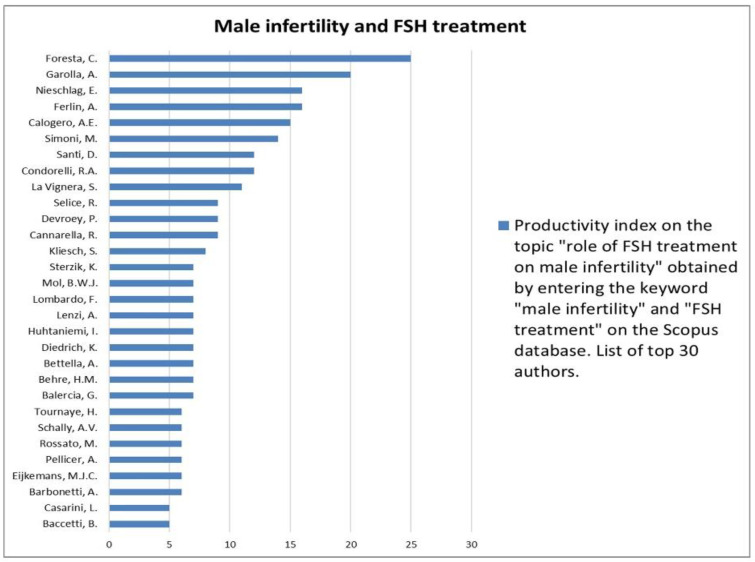
List of top 30 authors in terms of productivity regarding the association between male infertility and FSH treatment.

**Figure 22:**
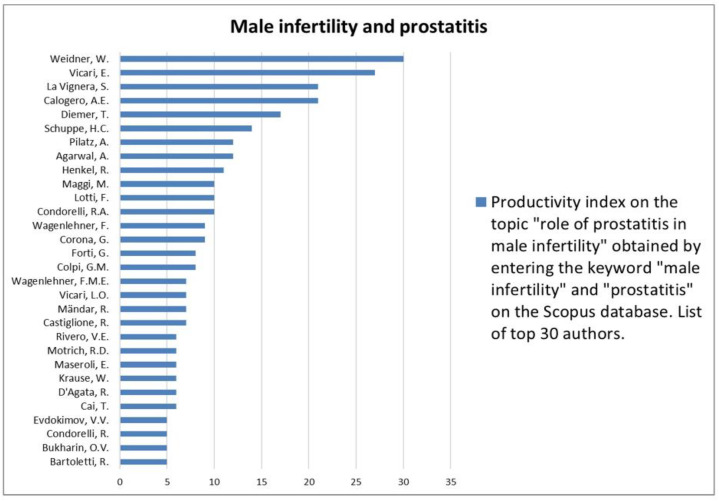
List of top 30 authors in terms of productivity regarding the association between male infertility and prostatitis.

**Figure 23:**
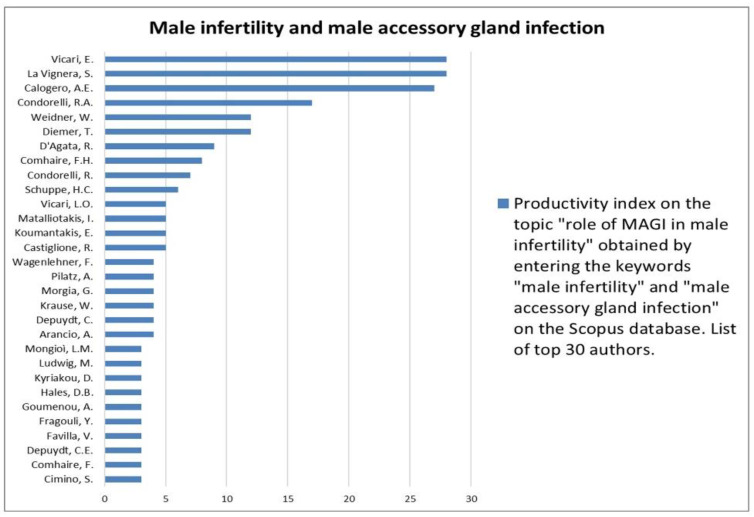
List of top 30 authors in terms of productivity regarding the association between male infertility and male accessory gland infection.

**Figure 24:**
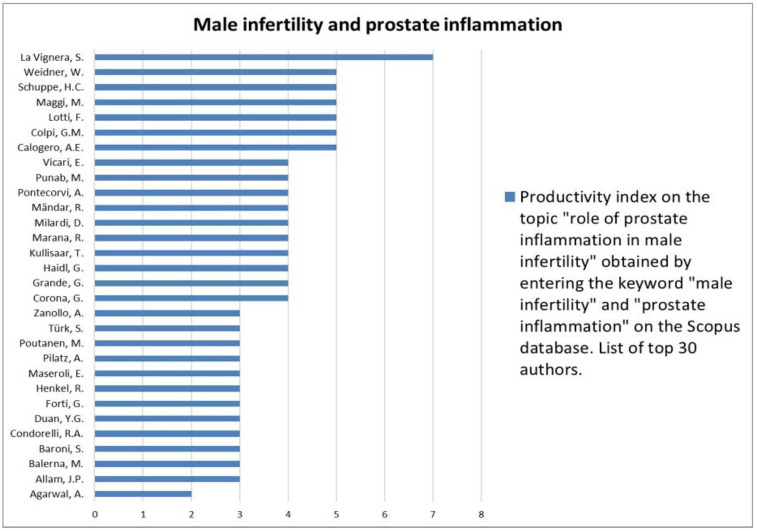
List of top 30 authors in terms of productivity regarding the association between male infertility and prostate inflammation.

**Table 1:** Ranking of the main andrological topics listed according to the number of publications (Scopus 21 April 2023).

Ranking Position	Topic	Number of Results
1	Prostate cancer	255,390
2	Male infertility	78,868
3	Erectile dysfunction	35,890
4	Prostatic hyperplasia	32,917
5	Testicular tumors	30,791
6	Male hypogonadism	17,782
7	Prostatitis	13,042
8	Prostate inflammation	9390
9	Varicocele	8796
10	Premature ejaculation	3678
11	Male accessory gland infection	315

**Table 2:** Ranking of the main andrological topics related to male infertility listed according to the number of publications (Scopus 21 April 2023).

Ranking Position	Topic	Number of Results
1	Male infertility and genetics	10,030
2	Male infertility and varicocele	3966
3	Male infertility and male hypogonadism	2527
4	Male infertility and sperm DNA fragmentation	2284
5	Male infertility and testicular cancer	1899
6	Male infertility and diabetes	1893
7	Male infertility and obesity	1884
8	Male infertility and FSH treatment	1358
9	Male infertility and prostatitis	628
10	Male infertility and male accessory gland infections	157
11	Male infertility and prostate inflammation	146

## Data Availability

Data available on request.
